# Socio-demographic correlates of ultra-processed food consumption in Canada

**DOI:** 10.1017/S1368980024001630

**Published:** 2024-09-26

**Authors:** Jane Y Polsky, Sara Jovovic, Milena Nardocci, Jean-Claude Moubarac

**Affiliations:** 1 Health Analysis Division, Statistics Canada, Ottawa, ON, Canada; 2 TRANSNUT (WHO Collaborating Centre on Nutrition Changes and Development), Département de Nutrition, Faculté de Médecine, Université de Montréal, Montréal, QC H3C 3J7, Canada; 3 Centre de Recherche en Santé Publique, Université de Montréal et CIUSS du Centre-Sud-de-l’île-de-Montréal, Montréal, QC, Canada

**Keywords:** Ultra-processed foods, Ultra-processing, Canada, Socio-demographic, Nutrition survey, NOVA

## Abstract

**Objective:**

To characterise consumption of ultra-processed foods and drinks (UPF) across a range of socio-demographic characteristics of Canadians.

**Design:**

Cross-sectional study. The national-level 2015 Canadian Community Health Survey–Nutrition provided data on all foods and drinks consumed on the previous day via a 24-hour dietary recall. All food items were classified according to the type of industrial processing using the NOVA system. Multivariable linear regression models examined associations between a range of socio-demographic characteristics and the mean energy contribution (% of total daily energy intake) from total UPF and UPF subgroups.

**Setting:**

The ten Canadian provinces.

**Participants:**

Canadians aged 2 or older (*n* 20 103).

**Results:**

UPF contributed, on average, nearly half (44·9 %) of total daily energy intake of Canadians. Children aged 6–12 and adolescents aged 13–18 consumed over half of total daily energy from UPF (adjusted means of 51·9 % and 50·7 %, respectively). Recent and long-term immigrants consumed a significantly lower share of energy from UPF (adjusted means of 42·2 % and 45·1 %, respectively) compared with non-immigrants (54·4 %), as did the food secure (42·8 %) *v*. those in moderately (48·1 %) or severely food-insecure households (50·8 %). More modest differences were observed for intake of total UPF and UPF subgroups by sex, education, income adequacy and region of residence.

**Conclusion:**

Levels of UPF consumption in 2015 in Canada were pervasive in all socio-demographic groups and highest among children and adolescents, non-immigrants and those living in food-insecure households. These findings can inform public health interventions to reduce UPF consumption and promote healthier diets in various socio-demographic groups.

Diet-related diseases and conditions, such as obesity, type 2 diabetes, hypertension and heart disease, are the leading cause of premature death around the world and their rates continue to increase^(1,2)^. The rise in prevalence of diet-related conditions co-occurred with changes in global dietary patterns, marked by a shift toward ultra-processed foods, eating out and lower intake of fruits and vegetables^(3,4)^. Such shifts have been observed in the Canadian diet as well: one analysis estimated that between 1938 and 2011, the replacement of whole and minimally processed foods and culinary ingredients with ready-to-eat and ready-to-heat ultra-processed products was one of the most important factors driving the shift in population dietary patterns^(5)^.

The industrialisation of food systems and technological changes have enabled the food industry to sell a larger variety and volume of ultra-processed food and drink products (UPF) in North America^(6)^. These products are industrial formulations of food substances, plus cosmetic additives like artificial flavours, colours and emulsifiers, that result from a series of industrial processes (hence ‘ultra-processed’)^(7)^. Typical examples include carbonated soft drinks, sweet or savoury packaged snacks, cookies and pastries, chocolate and candies (i.e. confectionery), reconstituted meat products and pre-prepared frozen dishes^(7)^. UPF are characterised by convenience (i.e. ready-to-eat or ready-to-heat), hyper-palatability, durability (i.e. long shelf life), attractive packaging and intense marketing^(7,8)^. They also tend to be highly profitable. UPF are defined as one of the four categories of the NOVA classification, a system that classifies foods and drinks according to the extent and purpose of processing^(7,8)^. The other three NOVA categories are unprocessed or minimally processed foods (e.g. fresh, dry and frozen fruits and vegetables; milk and plain yogurt; eggs; fresh and frozen meat and fish; pasta; grains and legumes) processed culinary ingredients (e.g. vegetable oils; butter; sugar; salt) and processed foods (e.g. canned fruits, vegetables and legumes; salted, cured and canned meat or fish; freshly made breads and cheeses)^(7,8)^.

There is a well-documented gradient in diet quality according to socio-economic position, such that individuals in lower socio-economic positions typically consume lower-quality diets^(9,10)^. Dietary patterns high in UPF tend to be of lower nutritional quality, namely energy-dense and high in nutrients of concern, including free sugars, saturated fat and sodium and low in fibre, vitamins and minerals^(11)^. Studies of UPF intake according to socio-demographic and socio-economic characteristics in high-income countries (e.g. UK, France, USA and Australia) have generally documented higher levels of UPF consumption among younger individuals, and those with lower levels of income and education, although patterns are not always consistent across socio-economic indicators and countries^(12–17)^. The handful of studies from high-income countries to examine UPF intake by immigrant status have generally observed lower intakes among the foreign-born *v*. those born in the host country^(13,18,19)^.

There is now consistent evidence from large-scale prospective studies from multiple countries that diets high in UPF are linked with elevated risk of diet-related conditions, including hypertension, CVD, type 2 diabetes, as well as premature mortality and mental health outcomes such as depression^(8,11,20,21)^. Given the serious negative diet- and health-related risks associated with high UPF consumption, identifying which socio-demographic groups, if any, consume higher levels of UPF could help inform policies and interventions to reduce disparities in diet quality and health outcomes.

Data from the most recent 2015 national-level dietary survey revealed that Canadians consumed, on average, nearly half of their total daily energy in the form of UPF^(22)^. Even though UPF represent a substantial share of Canadians’ total daily energy intakes, no previous study, to our knowledge, has conducted a focused investigation of whether UPF consumption varies across socio-demographic groups in Canada. Therefore, the objective of this study was to characterise the consumption of UPF across a range of socio-demographic characteristics of Canadians, namely, sex, age, education, income adequacy, immigrant status, household food security status and region of residence. This study uses the latest available population-level dietary data for Canada and presents data for children and adults.

## Methods

### Data source

Data for this study came from the 2015 Canadian Community Health Survey–Nutrition ^(23)^. The 2015 Canadian Community Health Survey–Nutrition has a sample size of 20 487 respondents, in which the target population were Canadian household residents aged one year or older living in the ten Canadian provinces. Full-time members of the Canadian Forces and individuals who lived on reserves or in other Indigenous settlements, in some remote areas, or in institutions were excluded from this study. The overall survey response rate was 61·6 %. The two components of the survey completed by all respondents were (1) a general demographic questionnaire and health questions and (2) a dietary recall of all foods and drinks consumed, including descriptions and amounts, in the 24 h prior to the interview day. About 30 % of participants completed a second 24-hour dietary recall 3–10 d after the initial interview; however, these data were not used in this study because group means from a single dietary recall represent unbiased estimates at the population level^(24)^.

Data were mainly collected in person by trained interviewers for the first recall and via telephone for the second recall. The Automated Multiple Pass Method adapted for Canada was used to help respondents maximise their dietary recall. For children under age 6, a parent or guardian provided information; for children 6–11, the interview was conducted with the child, with help from a parent; respondents aged 12 years and older provided their own information.

### Study sample

The present study sample was composed of Canadians aged 2 years or older who responded to the 2015 Canadian Community Health Survey–Nutrition. After excluding respondents younger than age 2 (*n* 372) and those who did not consume any energies on the previous day (*n* 12), the final analytic sample was 20 103 persons.

### Classification of food items according to NOVA

All food and drinks (excluding alcoholic drinks, which are not readily classifiable by NOVA) were categorised according to the NOVA classification system into four mutually exclusive groups: unprocessed or minimally processed foods; processed culinary ingredients; processed foods or UPF.^(7)^ The UPF group was further broken down into 13 subgroups. Classification of food items according to NOVA proceeded in two phases, following a previously described protocol^(22)^. Briefly, in the first phase, all ingredients, basic foods (i.e. foods that cannot be broken down into underlying ingredients like an apple or milk) and recipes without nutritional information available (e.g. some granola bars) were classified into one of four NOVA groups based on food item description. Energy values were based on the reported food amount converted into gram weight and were derived from the Canadian Nutrition File version 2015. In phase two, mixed dishes were searched to flag frozen meals, lunch kits and common ultra-processed dishes (e.g. burger, pizza and donut). If the flagged dish was consumed in a quick-service setting (e.g. pizza or fast-food restaurant), then all of its underlying ingredients were re-classified as UPF (subgroup ‘fast-food and frozen dishes’). For example, if a hamburger was consumed in a fast-food restaurant, then all of its underlying ingredients (e.g. bun, meat patty, tomato, lettuce, condiments) were re-classified as UPF. However, if the same hamburger was consumed at home, then phase one classification was maintained (i.e. bun and condiments categorised as UPF and meat and vegetables as processed or minimally processed).

### Measure of ultra-processed food consumption

Consumption of foods and drinks according to each of the four NOVA groups was defined as their relative energy contribution, i.e. the percentage of total daily energy intake (kcal) from each NOVA group or UPF subgroup.

### Socio-demographic variables

The choice of socio-demographic variables included in this study was informed by previous literature and data available on the survey. The socio-demographic variables included were sex (male or female), age (grouped as 2–5, 6–12, 13–18, 19–30, 31–54 or 55+ years) and the highest level of education attained in the household (less than high school, high school, certificate or diploma, or university degree or above). Household income adequacy (grouped into quintiles) was calculated as the adjusted ratio of total household income to the low-income cut-off corresponding to the household and community size. Immigrant status categorised respondents as either non-immigrant, recent immigrant (immigrated < 10 years ago), or long-term immigrant (immigrated to Canada 10 years ago or more). Household food security status was assessed using the Household Food Security Survey Module, which consists of eighteen questions that assess the income-related food security situation in the household in the previous 12 months. Respondents’ household food security status was categorised as food secure, moderately food insecure or severely food insecure. Region of residence was categorised as Atlantic provinces (New Brunswick, Nova Scotia, Prince Edward Island, Newfoundland and Labrador), Quebec, Ontario, Prairie provinces (Manitoba, Saskatchewan, Alberta) and British Columbia. Data for residents of the Atlantic and Prairie provinces were collapsed because of small sample sizes.

### Statistical analyses

The mean energy contribution (% of total daily energy intake) according to NOVA groups and UPF subgroups were generated overall and by respondent socio-demographic characteristics. Only the first 24-hour dietary recall was used to estimate the mean energy contributions from NOVA groups/subgroups. This is because mean intakes estimated from a single dietary recall are equivalent to usual (i.e. habitual) intakes at the population level^(24)^.

Associations between all socio-demographic characteristics and the mean energy contribution of UPF were assessed using multivariable linear regression models. Total UPF and each UPF subgroup were modelled separately. In addition, predicted mean energy contributions of UPF subgroups were generated for selected socio-demographic variables from the fully adjusted linear regression models. All analyses were conducted in SAS 9.4 and applied survey sampling weights provided by Statistics Canada to account for the complex sampling design and unequal probability of selection. Bootstrap weights were used to calculate robust se using the Balanced Repeated Replication method. Statistical significance was set at an alpha level of 0·05.

## Results

### Mean energy contribution from NOVA groups

In 2015, 40·6 % of total daily energy intake, on average, came from unprocessed or minimally processed foods and 7·1 % came from processed culinary ingredients (Table 1). Processed foods contributed 7·4 % of total daily energy and UPF contributed 44·9 %. Within UPF subgroups, most energies came from commercial breads, which contributed 10·4 % of total daily energy, followed by margarine (3·6 %), commercial fruit juices and drinks (3·6 %), and sauces, spreads and salad dressings (3·4 %).

**Table 1:** Mean energy contribution (% total daily energy) according to NOVA food groups and ultra-processed food and drink subgroups among Canadians aged 2 years and older, 2015 (*n* 20 103)

	% Total energy	95 % CI
		From, to
NOVA 1: Unprocessed or minimally processed foods	40·6	39·4, 41·8
NOVA 2: Processed culinary ingredients	7·1	6·9, 7·3
NOVA 3: Processed foods	7·4	7·1, 7·8
NOVA 4: Ultra-processed foods and drinks	44·9	43·6, 46·2
Bread, commercial	10·4	10·1, 10·7
Margarine	3·6	3·4, 3·8
Fruit juices and fruit drinks	3·6	3·4, 3·8
Sauces, spreads, salad dressings	3·4	3·2, 3·6
Fast-food and frozen dishes	3·3	2·8, 3·8
Sweetened milk- and soy-based products	3·1	2·9, 3·4
Chips, crackers, other salty snacks	2·9	2·7, 3·2
Chocolate and candies	2·9	2·7, 3·1
Cakes, cookies, other pastries	2·6	2·3, 2·8
Processed meat products	2·6	2·4, 2·7
Sweetened breakfast cereals	2·0	1·8, 2·1
Soft drinks	1·5	1·4, 1·7
Other*	3·1	2·8, 3·3

* ‘Other’ subgroup includes commercial soups, cheese products, baby products, meal replacements, imitation meat and fish, protein shake powder, protein bars, egg substitutes, instant coffee beverage and coffee substitutes, coffee whitener, sweeteners, etc.

### Sample characteristics

In the study sample, just over half of the respondents (50·7 %) were female and aged 31–54 years (50·1 %) (Table 2). Forty per cent of participants were in households where the highest level of education attained was a university degree or higher, followed by the certificate or diploma (37·4 %). Approximately three-quarters of participants (76·1 %) were non-immigrants to Canada, 7·1 % were recent immigrants (<10 years since immigration) and 16·8 % were long-term immigrants (10+ years since immigration). Household food insecurity (moderate or severe) was reported by 8·4 % of survey participants.

**Table 2:** Distribution (%) of the population and mean energy contribution (% of total daily energy) from NOVA food groups by socio-demographic characteristics among Canadians aged 2 years and older, 2015 (*n* 20 103)

		NOVA groups							
		NOVA 1. Unprocessed or minimally processed foods		NOVA 2. Culinary ingredients		NOVA 3. Processed foods		NOVA 4. Ultra-processed foods and drinks	
	Distribution	%	95 % CI	%	95 % CI	%	95 % CI	%	95 % CI
	%	Total energy	From, to	Total energy	From, to	Total energy	From, to	Total energy	From, to
Sex									
Male	49·3	40·0	38·6, 41·4	6·8	6·6, 7·0	7·2	6·6, 9·5	45·9	44·1, 48·8
Female	50·7	41·1	39·9, 42·3	7·3	6·9, 7·7	7·6	7·2, 9·8	43·9	42·9, 46·4
Age (years)									
2–5	4·7	41·7	39·9, 43·5	4·1	3·7, 4·5	6·5	5·7, 8·9	47·8	46·0, 50·7
6–12	7·5	35·9	34·7, 37·1	5·2	4·8, 5·6	6·2	5·6, 8·5	52·7	51·3, 55·4
13–18	6·7	36·6	35·2, 38·0	5·4	5·0, 5·8	6·9	6·1, 9·3	51·0	49·6, 53·7
19–30	13·6	40·9	38·7, 43·1	7·1	6·5, 7·7	7·3	6·3, 9·8	44·7	42·3, 47·9
31–54	50·1	41·7	39·9, 43·5	7·6	7·2, 8·0	8·0	7·4, 10·3	42·7	40·9, 45·6
55+	17·3	40·4	38·2, 42·6	7·9	7·3, 8·5	6·8	6·2, 9·1	44·9	42·2, 48·3
Immigrant status									
Non-immigrant	76·1	37·9	37·3, 38·5	6·9	6·7, 7·1	7·2	7·0, 9·3	48·0	47·4, 50·3
Long-term immigrant (10+ years)	16·8	48·0	45·8, 50·2	7·7	7·1, 8·3	8·3	7·1, 10·9	36·1	33·6, 39·4
Recent immigrant (< 10 years)	7·1	50·8	48·8, 52·8	7·5	6·7, 8·3	8·4	7·0, 11·1	33·3	31·5, 36·2
Education									
< High school	6·2	38·5	36·5, 40·5	7·4	6·8, 8·0	5·1	3·9, 7·7	49·0	46·8, 52·1
High school	16·4	38·7	37·1, 40·3	7·0	6·4, 7·6	6·9	5·9, 9·4	47·4	45·4, 50·4
Certificate or diploma	37·4	38·6	37·0, 40·2	6·8	6·4, 7·2	7·7	6·7, 10·2	46·8	45·8, 49·3
University degree	40·1	43·5	42·3, 44·7	7·3	6·9, 7·7	7·7	6·9, 10·1	41·4	39·6, 44·3
Income adequacy									
Quintile 1 (lowest)	19·9	42·0	40·2, 43·8	6·8	6·2, 7·4	7·3	6·5, 9·7	43·8	41·8, 46·8
Quintile 2	20·3	40·6	38·6, 42·6	7·1	6·7, 7·5	7·3	6·5, 9·7	45·1	42·7, 48·3
Quintile 3	20·4	40·5	39·1, 41·9	7·1	6·5, 7·7	7·0	5·8, 9·6	45·3	43·5, 48·2
Quintile 4	19·6	40·2	38·8, 41·6	7·6	6·6, 8·6	7·7	6·9, 10·1	44·6	43·2, 47·3
Quintile 5 (highest)	19·8	39·7	38·5, 40·9	6·8	6·2, 7·4	7·8	7·2, 10·1	45·7	44·1, 48·5
Household food security status									
Food secure	91·6	40·9	39·7, 42·1	7·1	6·9, 7·3	7·6	7·2, 9·8	44·3	43·1, 46·9
Moderately food insecure	5·9	37·3	33·8, 40·8	6·2	5·2, 7·2	6·1	4·7, 8·8	50·4	45·5, 54·9
Severely food insecure	2·5	35·0	32·3, 37·7	7·3	6·1, 8·5	4·4	3·0, 7·1	53·3	50·2, 56·9
Region of residence									
Atlantic provinces	6·6	35·9	34·9, 36·9	7·0	6·4, 7·6	6·2	5·8, 8·4	50·9	49·5, 53·6
Quebec	23·2	39·4	38·0, 40·8	7·2	6·6, 7·8	7·3	6·7, 9·6	46·1	44·7, 48·8
Ontario	38·9	41·6	39·2, 44·0	7·2	6·8, 7·6	7·6	6·8, 10·0	43·6	41·1, 46·9
Prairie provinces	18·1	39·3	37·9, 40·7	6·6	6·2, 7·0	7·6	7·0, 9·9	46·5	45·1, 49·2
British Columbia	13·1	44·0	42·6, 45·4	7·1	6·7, 7·5	7·5	6·7, 9·9	41·4	39·8, 44·2

### Mean energy contribution according to NOVA by socio-demographic characteristics

Table 2 also shows the mean energy contribution (% of total daily energy) according to NOVA groups by socio-demographic characteristics. There was more variation across several socio-demographic groups in the mean percentage of energy from unprocessed or minimally processed foods and UPF than from culinary ingredients and processed foods. The mean percentage of energy from unprocessed or minimally processed foods ranged from 35·0 % among respondents living in households with severe food insecurity to 50·8 % among recent immigrants. Conversely, the energy contribution from UPF was lowest among recent immigrants (33·3 %) and highest among the severely food insecure (53·3 %). There was also some variation across age groups, education level and region of residence, but little variation by sex and household income adequacy.

### Associations between socio-demographic characteristics and ultra-processed food consumption

Tables 3(a) and (b) present mutually adjusted associations of socio-demographic characteristics with total UPF consumption (mean % of total daily energy) and subgroups of UPF. For total UPF consumption, there were statistically significant associations for all socio-demographic characteristics examined. For example, compared to children aged 2–5 years, children aged 6–12 years on average consumed 5·3 percentage points more total daily energy from UPF (*β* = 5·3; *P*-value < 0·0001), while adults aged 31–54 consumed 2·7 fewer percentage points (*β* = –2·7; *P*-value < 0·0001). Differences in total UPF consumption were most notable between immigrants *v*. non-immigrants and between those living in food-secure *v*. food-insecure households.

**Table 3:** (a) Associations between socio-demographic characteristics and percentage of total daily energy from total ultra-processed foods and drinks (UPF) and UPF subgroups, Canadians aged 2 and older, 2015 (*n* 20 103)

	Total UPF		Bread, commercial		Margarine		Fruit juices & fruit drinks		Sauces, spreads, salad dressings		Fast-food & frozen dishes		Sweetened milk- & soy-based products	
	*β*	95 % CI	*β*	95 % CI	*β*	95 % CI	*β*	95 % CI	*β*	95 % CI	*β*	95 % CI	*β*	95 % CI
		From, to		From, to		From, to		From, to		From, to		From, to		From, to
Sex														
Male	Ref.		Ref.		Ref.		Ref.		Ref.		Ref.		Ref.	
Female	−1·7***	−2·7, –0·7	−0·3	−0·9, 0·3	−0·3	−0·5, 0·0	−0·3	−0·7, 0·2	0·1	−0·1, 0·4	−0·9***	−1·4, –0·4	0·6*	0·1, 1·1
Age (years)														
2–5	Ref.		Ref.		Ref.		Ref.		Ref.		Ref.		Ref.	
6–12	5·3***	3·1, 7·4	1·7*	0·1, 3·3	0·2	−0·5, 0·8	0·0	−0·7, 0·7	−0·1	−0·8, 0·5	0·4	−0·4, 1·1	−0·3	−1·1, 0·6
13–18	4·1***	1·8, 6·3	0·3	−0·7, 1·4	0·3	−0·4, 1·0	−0·2	−0·9, 0·5	0·8*	0·1, 1·4	2·0**	0·8, 3·2	−1·3**	−2·2, –0·5
19–30	−1·7	−4·8, 1·4	0·5	−1·1, 2·1	0·4	−0·1, 0·9	−0·9*	−1·8, 0·0	1·5***	0·6, 2·4	2·4	0·0, 4·9	−2·6***	−3·5, –1·7
31–54	−2·7**	−4·7, –0·8	1·9***	1·0, 2·9	0·3	−0·4, 0·9	−2·6***	−3·1, –1·9	1·2**	0·3, 2·1	2·2***	1·1, 3·4	−2·5***	−3·2, –1·8
55+	−1·3	−3·7, 1·0	4·1***	3·0, 5·2	1·3***	0·8, 1·7	−2·6***	−3·4, –1·7	1·0**	0·3, 1·7	0·7	−0·2, 1·5	−2·0***	−2·8, –1·3
Education														
< High school	Ref.		Ref.		Ref.		Ref.		Ref.		Ref.		Ref.	
High school	−1·1	−3·3, 1·2	0·0	−1·3, 1·4	−0·1	−0·8, 0·6	0·0	−0·8, 0·8	0·1	−0·4, 0·6	0·4	−0·9, 1·7	0·0	−1·0, 0·9
Certificate or diploma	−1·6	−3·7, 0·4	−0·7	−2·4, 1·0	−0·4	−1·1, 0·2	−0·3	−1·2, 0·5	0·5	−0·3, 1·2	0·4	−0·8, 1·5	0·2	−0·9, 1·3
University degree	−5·7***	−8·0, –3·4	−1·2	−2·4, 0·0	−0·6*	−1·1, –0·1	−0·3	−1·1, 0·5	−0·4	−0·9, 0·1	0·0	−1·1, 1·2	0·3	−0·9, 1·5
Income adequacy														
Quintile 1 (lowest)	Ref.		Ref.		Ref.		Ref.		Ref.		Ref.		Ref.	
Quintile 2	1·8	−0·3, 4·0	−0·1	−1·1, 0·9	−0·1	−0·4, 0·2	−0·2	−1·2, 0·8	0·3	−0·1, 0·7	0·5	−0·4, 1·5	0·1	−0·5, 0·6
Quintile 3	2·0	−0·4, 4·4	−0·4	−1·6, 0·8	0·4	0·0, 1·8	0·1	−0·7, 0·8	0·4	−0·4, 1·2	−0·2	−1·0, 0·6	0·3	−0·3, 0·8
Quintile 4	1·5	−0·4, 3·4	−0·3	−1·3, 0·7	0·4	−0·3, 1·1	−0·5	−1·7, 0·7	0·6	−0·1, 1·4	−0·7	−1·8, 0·5	0·2	−0·4, 0·8
Quintile 5 (highest)	2·8**	0·7, 4·8	−0·3	−1·3, 0·8	0·4	−0·3, 1·0	−0·4	−2·0, 1·3	0·9	0·0, 1·9	0·8	−0·6, 2·1	0·2	−0·4, 0·8
Immigrant status														
Non-immigrant	Ref.		Ref.		Ref.		Ref.		Ref.		Ref.		Ref.	
Long-term immigrant (10+ years)	−9·3***	−11·8, –6·9	−0·1	−1·3, 1·0	0·0	−0·4, 0·3	0·1	−0·5, 0·8	−1·5***	−2·0, –1·1	−1·6*	−2·8, –0·3	−0·7*	−1·4, –0·1
Recent immigrant (<10 years)	−12·2***	−14·1, –10·3	−1·5*	−2·7, –0·3	0·4	−0·3, 1·2	−0·2	−0·9, 0·4	−1·4***	−1·8, –0·9	−2·6***	−4·0, –1·2	−1·3***	−1·9, –0·7
Household food security status														
Food secure	Ref.		Ref.		Ref.		Ref.		Ref.		Ref.		Ref.	
Moderately food insecure	5·4**	1·4, 9·4	1·3	−0·9, 3·5	0·1	−0·5, 0·7	0·4	−0·4, 1·2	0·7*	0·1, 1·2	0·0	−0·9, 1·0	−0·2	−1·2, 0·8
Severely food insecure	8·1***	4·3, 11·9	1·3	−1·6, 4·2	0·9	−0·2, 2·0	−0·6	−3·4, 2·1	0·6	−0·3, 1·5	1·4	−1·2, 4·0	−0·9**	−1·5, –0·3
Region of residence														
Atlantic provinces	Ref.		Ref.		Ref.		Ref.		Ref.		Ref.		Ref.	
British Columbia	−6·1***	−8·2, –4·0	−1·2**	−2·1, –0·4	−0·7***	−1·1, –0·4	−0·5	−1·1, 0·1	0·4	−0·7, 1·5	−0·4	−1·2, 0·4	−0·4	−0·9, 0·2
Ontario	−4·1***	−5·6, –2·6	−0·8	−2·2, 0·6	−0·6***	−0·9, –0·2	−0·3	−0·8, 0·3	0·0	−0·5, 0·5	0·8	−0·3, 1·9	−0·2	−0·8, 0·5
Prairie provinces	−2·9***	−4·5, –1·3	−1·4*	−2·7, –0·2	−0·7***	−1·0, –0·4	−0·1	−0·9, 0·6	−0·4*	−0·8, –0·1	1·0**	0·3, 1·8	−0·2	−0·8, 0·4
Quebec	−2·8***	−4·4, –1·2	0·2	−0·7, 1·1	−0·7	−1·4, 0·1	0·7**	0·2, 1·2	0·4	−0·1, 0·8	−0·7	−1·5, 0·0	−0·3	−0·8, 0·2

Ref., referent group; UPF, ultra-processed foods and drinks.

Parameter estimates (*βs)* and associated 95 % CI were calculated using multivariable linear regression models and adjusted for all socio-demographic covariates listed in the table. Total UPF and each UPF subgroup were modelled separately.

Estimates are significantly different from those of the referent group: *P < 0·05, **P < 0·01, ***P < 0·001.

Mutually adjusted associations of socio-demographic characteristics and the mean percentage of energy derived from 13 subgroups of UPF revealed differences across several characteristics, most notably, age group, immigrant status and region of residence (Tables 3(a) and (b)). The relative energy contribution of UPF subgroups varied substantially by age group. For example, soft drinks contributed, on average, substantially more relative energy for children, adolescents and adults under age 55 compared with the youngest children aged 2–5, while fruit juices and fruit drinks contributed significantly less energy for adults. Notable differences between adults and children were also seen for relative energy from sauces, spreads and salad dressings; sweetened milk and soy-based products; cakes, cookies and other pastries; and sweetened breakfast cereals. Fast-food and frozen dishes contributed significantly more relative energy for adolescents and younger adults, and commercial breads and margarine contributed more relative energy for older adults aged 55+ compared to the youngest children.

**Table 3:** (b) Associations between socio-demographic characteristics and percentage of total daily energy from ultra-processed food and drink subgroups, Canadians aged 2 and older, 2015 (*n* 20 103)

	Chips, crackers, other salty snacks		Chocolate & candies		Cakes, cookies, other pastries		Processed meat products		Sweetened breakfast cereals		Soft drinks		Other	
	*β*	95 % CI	*β*	95 % CI	*β*	95 % CI	*β*	95 % CI	*β*	95 % CI	*β*	95 % CI	*β*	95 % CI
		From, to		From, to		From, to		From, to		From, to		From, to		From, to
Sex														
Male	Ref.		Ref.		Ref.		Ref.		Ref.		Ref.		Ref.	
Female	0·1	−0·4, 0·6	0·7**	0·4, 1·0	0·1	−0·3, 0·5	−1·1***	−1·3, –0·8	0·1	−0·4, 0·5	−0·7***	−0·9, –0·5	0·1	−0·3, 0·4
Age (years)														
2–5	Ref.		Ref.		Ref.		Ref.		Ref.		Ref.		Ref.	
6–12	1·5*	0·5, 2·4	1·3*	0·6, 1·9	0·6	0·0, 1·1	−0·2	−0·9, 0·5	0·1	−0·4, 0·6	0·8***	0·6, 1·0	−0·6	−1·2, 0·1
13–18	0·5	0·0, 0·9	0·4	−0·4, 1·2	−0·1	−0·6, 0·5	−0·4	−0·8, 0·1	−0·1	−0·6, 0·5	2·1***	1·9, 2·2	−0·2	−0·6, 0·3
19–30	−0·5	−1·2, 0·1	−1·5**	−2·0, –1·1	−1·3*	−2·0, –0·7	−0·1	−0·6, 0·4	−1·2**	−1·7, –0·7	1·9**	1·2, 2·7	−0·3	−0·8, 0·2
31–54	−0·4	−1·4, 0·5	−1·2	−2·1, 0·0	−1·8***	−2·3, –1·3	−0·4	−1·2, 0·3	−1·1***	−1·4, –0·8	1·7***	1·4, 2·1	−0·1	−0·5, 0·4
55+	−1·2*	−2·0, –0·4	−1·2	−2·1, 0·0	−1·6**	−2·3, –0·9	−0·1	−1·0, 0·7	−0·3	−0·9, 0·2	1·0**	0·6, 1·3	−0·2	−0·8, 0·4
Education														
< High school	Ref.		Ref.		Ref.		Ref.		Ref.		Ref.		Ref.	
High school	−0·4	−1·6, 0·9	0·3	−0·3, 0·9	−0·3	−1·2, 0·6	−0·7	−2·0, 0·6	−0·1	−0·8, 0·7	0·0	−0·9, 0·9	−0·3	−0·7, 0·2
Certificate or diploma	−0·5	−1·3, 0·4	0·5	0·0, 1·0	−0·1	−0·8, 0·6	−0·5	−1·7, 0·7	0·1	−0·5, 0·8	−0·3	−1·3, 0·7	−0·6*	−1·0, –0·2
University degree	−0·8	−1·7, 0·1	0·5	0·0, 1·1	−0·1	−0·8, 0·6	−1·4	−2·7, 0·0	0·1	−0·7, 0·8	−0·7	−1·8, 0·5	−1·1**	−1·6, –0·6
Income adequacy														
Quintile 1 (lowest)	Ref.		Ref.		Ref.		Ref.		Ref.		Ref.		Ref.	
Quintile 2	0·4	−0·2, 0·9	0·1	−0·9, 1·1	0·1	−0·4, 0·6	0·2	0·0, 0·3	0·3	0·0, 0·5	0·1	−0·4, 0·6	0·2	−0·4, 0·8
Quintile 3	0·6	0·0, 1·2	0·1	−0·7, 0·8	−0·1	−0·4, 0·2	0·5*	0·2, 0·9	0·2	−0·1, 0·5	0·1	−0·5, 0·8	0·0	−0·3, 0·2
Quintile 4	0·3	−0·9, 1·6	0·2	−1·0, 1·5	0·0	−0·5, 0·5	0·9***	0·7, 1·1	0·3	−0·3, 0·8	0·2	−0·5, 0·8	−0·2	−1·0, 0·6
Quintile 5 (highest)	0·7*	0·2, 1·1	−0·4	−1·0, 0·2	0·1	−0·7, 0·8	1·0	0·0, 1·8	0·3	−0·1, 0·6	−0·3	−0·7, 0·2	−0·2	−1·0, 0·6
Immigrant status														
Non-immigrant	Ref.		Ref.		Ref.		Ref.		Ref.		Ref.		Ref.	
Long-term immigrant (10+ years)	−1·6***	−2·1, –1·2	−0·4	−1·0, 0·3	−0·8**	−1·1, –0·4	−0·6	−1·5, 0·2	−0·5*	−0·8, –0·3	−0·9***	−1·1, –0·7	−0·6	−1·6, 0·4
Recent immigrant (<10 years)	−1·4**	−1·9, –0·9	−0·6	−1·5, 0·3	−0·4	−1·2, 0·4	−1·1***	−1·4, –0·9	−0·7	−1·4, 0·0	−0·5	−0·9, 0·0	−1·0***	−1·1, –0·8
Household food security status														
Food secure	Ref.		Ref.		Ref.		Ref.		Ref.		Ref.		Ref.	
Moderately food insecure	0·4	−1·6, 2·4	−0·1	−0·7, 0·6	0·4	−0·2, 1·1	0·7	−0·4, 1·7	0·0	−0·1, 0·2	1·1	0·2, 1·9	0·6	0·0, 1·1
Severely food insecure	0·6	−0·9, 2·2	0·9	−0·3, 2·2	0·4	−0·6, 1·3	0·9	−0·3, 2·0	−0·3	−1·2, 0·6	1·4*	0·5, 2·4	1·5	−1·1, 4·1
Region of residence														
Atlantic provinces	Ref.		Ref.		Ref.		Ref.		Ref.		Ref.		Ref.	
British Columbia	−0·9	−1·9, 0·1	−0·7	−1·5, 0·0	−0·3	−0·8, 0·1	−0·3	−0·5, 0·0	−0·1	−0·4, 0·2	−1·1*	−1·7, –0·4	0·1	−0·6, 0·9
Ontario	−0·7	−1·4, 0·1	−1·0**	−1·3, –0·6	−0·2	−0·6, 0·1	−0·4	−0·8, 0·0	−0·2	−0·5, 0·1	−0·5*	−0·8, –0·1	−0·2	−0·7, 0·3
Prairie provinces	−0·4	−0·7, 0·0	−0·4	−1·0, 0·2	−0·5*	−0·9, –0·2	−0·4*	−0·6, –0·2	−0·2	−0·4, 0·0	−0·2	−0·5, 0·2	1·0	0·0, 2·1
Quebec	−1·3*	−2·0, –0·7	−0·6	−1·6, 0·3	−0·1	−0·6, 0·4	0·4	−0·1, 0·9	0·0	−0·5, 0·6	−0·8***	−1·0, –0·6	0·0	−0·3, 0·3

Ref., referent group.

Parameter estimates (*βs)* and associated 95 % CI were calculated using multivariable linear regression models and adjusted for all socio-demographic covariates listed in the table. Each ultra-processed subgroup was modelled separately.

Estimates are significantly different from those of the referent group: *P < 0·05, **P < 0·01, ***P < 0·001.

Immigrants reported consistently lower shares of energy from virtually all UPF subgroups than non-immigrants, particularly from sauces, spreads and salad dressings; fast-food and frozen dishes; and chips, crackers and other salty snacks. Among recent immigrants, the mean energy contribution from commercial breads, sweetened milk- and soy-based products, processed meat products and ‘other’ UPF (e.g. meal replacements, protein shakes, sweeteners, imitation fish/meats) was notably lower than among non-immigrants.

For region of residence, residents of Atlantic provinces consumed a higher share of energy from nearly all UPF subgroups compared to residents of other regions, although differences were not always statistically significant.

There were some differences between males and females in the mean energy contribution from UPF subgroups, particularly for processed meat products (*β* = –1·1 for females *v*. males; *P*-value < 0·001). For household food security status, the share of energy from UPF subgroups was typically higher among those in food-insecure households *v*. food-secure ones, although few differences were statistically significant. Few differences were seen across household education or income adequacy strata.

### Adjusted mean ultra-processed food consumption

**Fig. 1:**
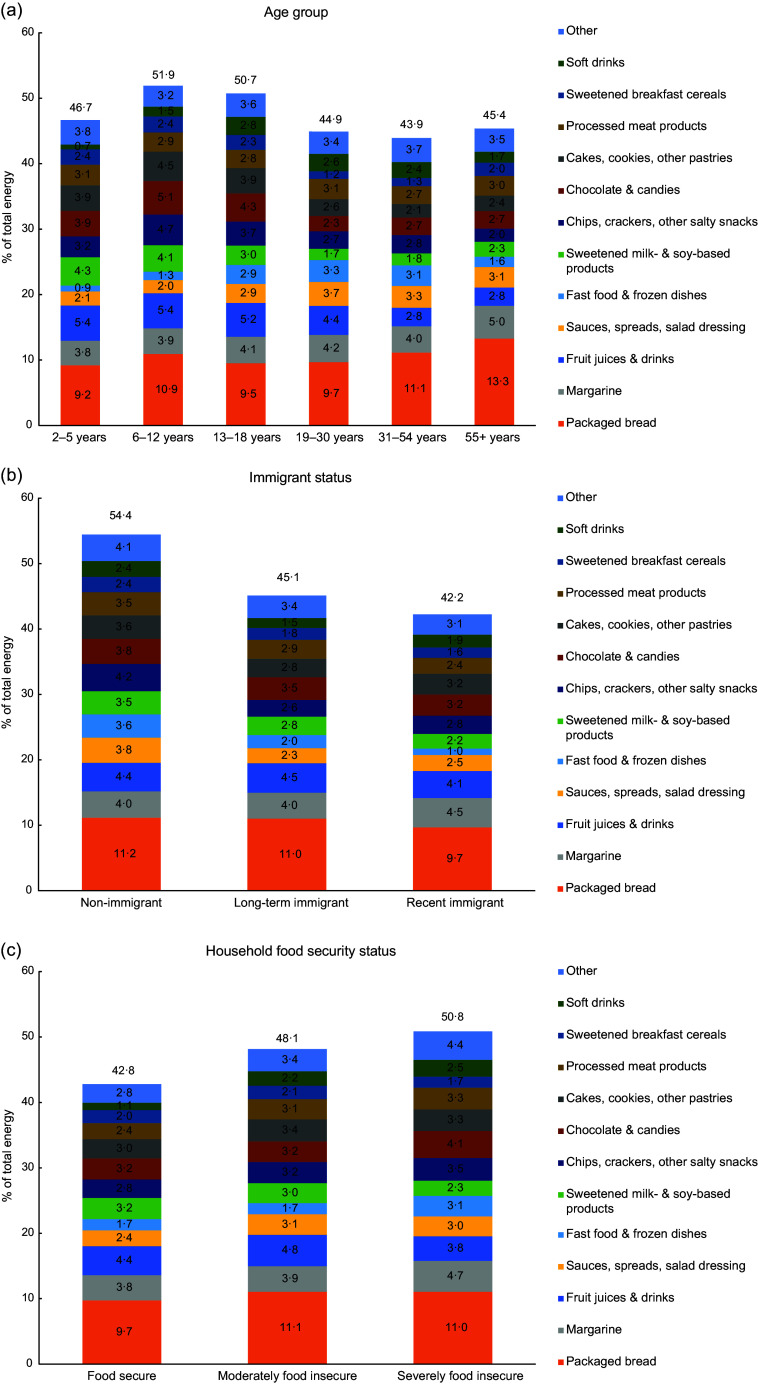
Adjusted mean percentage^†^ of total daily energy from ultra-processed food subgroups for selected socio-demographic characteristics: (a) age group, (b) immigrant status and (c) household food security status. † Means are predicted means from linear regression models mutually adjusted for all socio-demographic covariates under study (i.e. age, sex, education, income adequacy, immigrant status, household food security status and region of residence). Each ultra-processed food subgroup was modelled separately. ‘Recent immigrant’ was defined as < 10 years since immigration and ‘long-term immigrant’ as 10+ years since immigration

As a complement to the multivariable linear regression results shown in Tables 3(a) and (b), Fig. 1 panels (a) through (c) present the predicted mean percentages of total energy derived from UPF subgroups for selected socio-demographic variables, adjusted for all other socio-demographic covariates under study. Children aged 6–12 and adolescents aged 13–18 consumed over half of total daily energy from UPF (adjusted means of 51·9 % and 50·7 %, respectively; Fig. 1, panel A). The largest absolute differences in the mean percentage of energy from UPF (of at least 9 percentage points), adjusted for all other socio-demographic variables, were observed between recent and long-term immigrants *v*. non-immigrants (Fig. 1, panel B).

## Discussion

This study used data from the most recently available national-level dietary survey (2015) to examine whether consumption of UPF varies across a range of socio-demographic characteristics of Canadian children and adults. Results of multivariable analyses revealed that while consumption of UPF in Canada was pervasive, there was some variation in the mean energy contribution of UPF and subgroups of UPF across all socio-demographic characteristics examined. Higher levels of UPF consumption were documented among males, children and youth, persons in households with less than high school education, households with higher-income adequacy, non-immigrants and those living in food-insecure households and in the Atlantic region of the country. The most substantive differences in the mean energy contribution of UPF were observed by age group, immigrant status and household food security status.

This study documented a slightly higher energy share of total UPF consumption among males than females, adjusted for all other socio-demographic characteristics under study, which is consistent with some previous studies from high-income countries^(14,25)^ but not others^(13,15,17,26)^. In this study, females consumed less fast-food and frozen dishes, processed meats and soft drinks than males, but more sweetened milk and soy-based products, and chocolate and candies. These findings likely reflect gender roles and identities around household food shopping and preparation^(27)^, as well as lower frequency of eating out among Canadian women, particularly older women^(28)^. Given the limited literature on sex/gender differences in UPF consumption, future studies should further explore these patterns using more recent dietary data, particularly using qualitative methods, and investigate how sex/gender is leveraged in marketing strategies.

There were notable differences in UPF consumption by age group. Children and youth aged 6–18 were the highest consumers of UPF, deriving on average 50 % or more total energy from UPF. This finding echoes reports from multiple countries including the UK, Italy, USA and Australia, which consistently document the highest levels of UPF intake among children and youth^(17,25)^ or younger adults^(13,14,16,26)^. The negative association of UPF intake with age could be shaped by differences in age-specific food preferences (e.g. young children consumed more fruit juice/drinks and cakes, cookies and other pastries than other age groups, while adolescents and younger adults consumed more fast-food and frozen dishes, and soft drinks). These preferences may be driven by children and young people’s increased nutritional requirements, peer social pressure, limited cooking skills and affordability of UPF^(8,29,30)^. UPF are both extensively marketed to younger persons and highly available in the various settings where children and youth live, learn and play (e.g. in schools and recreational sports facilities)^(31)^. Younger adults may be early adopters of new energy-dense products available in their food environments (e.g. school snacks)^(32)^ and consume a higher diversity of UPF products than older adults^(26)^. Conversely, older adults may consume less UPF because of lack of familiarity with many UPF products and/or because of health concerns^(26)^.

This study found relatively high levels of UPF consumption across the spectrum of household income adequacy and educational attainment, although with some diverging patterns: total UPF intake was slightly higher among individuals in the top quintile of household income adequacy *v*. the lowest and modestly lower among individuals in households with a university degree or above *v*. those with less than high school education. There were few differences across UPF subgroups for either variable. Our results are highly consistent with a multi-year national-level study of US adults (2001–2018), which similarly documented little difference in UPF consumption according to family income-to-poverty ratio and lower intake among college graduates^(33)^. Studies from multiple countries similarly report inconsistent patterning of UPF consumption by level of education and income^(12)^. This is not unexpected if one considers that household income typically reflects direct access to material resources (i.e. economic capital), whereas educational attainment indirectly captures access to material resources via occupation and income, in addition to capturing non-material resources like social and cultural capital (e.g. health consciousness, nutrition literacy and perceived diet-related norms)^(9,34,35)^. As such, social and cultural capital accumulated by highly educated individuals may unwittingly direct them to adopt ‘healthier’, less processed diets (independent of material resources), possibly as a means of distinguishing themselves from those with lower levels of education^(36,37)^. Further, it is possible that individuals in different income strata consume different types of UPF products. The increasingly available ‘premium’ UPF are marketed as higher quality and with functional properties for targeted health (e.g. immune boost) and environmental benefits (e.g. plant-based meats)^(38)^. Such products tend to cost more than traditional UPF^(38)^, making them more accessible for higher-income *v*. lower-income households. While this study’s dietary data did not allow to distinguish between premium and more traditional UPF, future studies should explore any differential patterns in the purchase and consumption of various types of UPF by income and education as distinct socio-economic indicators.

This study is among the few to examine UPF consumption in relation to income-related household food insecurity (i.e. inadequate or insecure access to food because of financial constraints). In line with a handful of previous reports from Canada and the USA,^(39,40)^ we documented that individuals living in food-insecure households consumed substantially more energy from UPF than those in food-secure households. While income-related food insecurity is inextricably linked with household income, these measures involve notable distinctions. Household income adequacy is an objective measure of household income-to-poverty ratio that doesn’t capture factors like savings, assets or debt. In contrast, food insecurity is a subjective measure of household financial ability to ensure secure access to food in the previous year. In addition to compromises in food quality and/or quantity, food-insecure households make trade-offs in multiple spheres of life like housing and bill payments.^(41)^ Food insecurity may therefore provide a more comprehensive depiction of household financial circumstances, including the ability to weather negative income shocks like unexpected expenses^(40,41)^.

As such, food insecurity is a highly sensitive marker of financial precarity – over and above household income – and has been consistently linked with poorer diet quality and a range of adverse health outcomes^(40,42)^. Higher intake of UPF among those experiencing income-related food insecurity in this study is consistent with North American evidence that diets high in UPF typically have low per-energy cost^(43)^ and that food-insecure households spend less on food than the food secure^(41)^. Households are known to use multiple strategies to cope with food insecurity (e.g. buying foods on sale), some of which can reinforce UPF consumption. For example, food-insecure families with children have reported keeping more UPFs like microwavable or quick-cook frozen meals in their kitchens, decreasing consumption of some unprocessed or minimally processed foods (e.g. meat, eggs, beans, fruits and vegetables) or increasing consumption of other foods like grains and starches (e.g. noodles) and mixed dishes (e.g. sandwiches) as strategies to mitigate food hardship^(44,45)^. Given the paucity of research on the topic, future studies are needed to better understand patterns and correlates of UPF consumption in the context of food insecurity.

Immigrant status was the most potent predictor of UPF consumption in this study, after accounting for other socio-demographic characteristics. Both recent and long-term immigrants reported consuming substantially less UPF than Canadian-born individuals. Similar patterns were documented in national-level data from the USA and Australia^(13,19)^. These results are consistent with the healthy immigrant effect, which postulates that immigrants have better health behaviours prior to arriving in the host country because good health is typically a prerequisite to immigration^(46)^. Compared to those born in Canada, this study found that immigrants consumed less energy from several UPF subgroups, including fast-food and frozen dishes; sauces, spreads and salad dressings; and salty snacks. Such differences may be explained by evidence that foreign-born individuals living in North America tend to prepare more meals at home^(47)^, possibly as a means of preserving their culinary traditions, or because they may not have become fully familiarised with ‘Western’ dietary pattern, which is typically high in UPF.

However, over time, immigrants tend to undergo the process of acculturation whereby they adopt the dietary practices of the host country. In the USA, acculturation among immigrants was positively associated with UPF consumption and poor diet quality^(18,19)^. Qualitative studies would help to better understand how immigrants perceive UPF and the context in which they consume these foods. The current study’s findings highlight the need for programmes and strategies to slow down the dietary acculturation process in order to prevent the deterioration of immigrants’ diet quality and health. This can include enhanced support for and expansion of existing strategies to promote and protect various ethnic cuisines of immigrant communities, such as initiatives within the Canada’s federal Healthy Eating Strategy^(48)^.

### Study limitations

This study is strengthened by the use of a large, national-level sample and examination of a range of socio-demographic characteristics. However, several limitations deserve mention. During the classification of food items into NOVA groups, some foods and drinks may have been misclassified because of the lack of details available about product brands and type of food processing. For example, some mixed dishes should be disaggregated into underlying ingredients and classified as minimally processed if they were prepared at home from scratch ingredients *v*. ultra-processed if prepared industrially (e.g. pizza). In this study, such dishes were classified as UPF only if they were consumed in a quick-service setting (e.g. a fast-food restaurant) but not if consumed outside of the food service setting (e.g. as takeout or delivery) because information on the type of food processing and place of food preparation was not consistently available. This may have attenuated the level of UPF consumption and the associations under study. Furthermore, social desirability bias may have caused underreporting of certain foods (e.g. fast foods, salty snacks, soft drinks), leading to underestimation of UPF consumption. If this misreporting differed across socio-demographic groups, this could have led to either underestimation or overestimation of the associations under study. While data from a single 24-hour dietary recall are useful for estimating group means, as was done in this study, they do not fully capture all intra-person variability and thus may not represent the usual dietary intakes of individuals. Further, as with all observational research, we cannot rule out the presence of residual confounding by unmeasured or mismeasured characteristics. Finally, the results are based on data collected in 2015. While these data represent the most recently available national-level nutrition survey data for Canada, it will be important to examine any changes in socio-economic patterning of UPF consumption using more recent data, particularly following the release of an updated Canada Food Guide in 2019^(49)^ and the rising cost of living since 2021^(50)^.

### Conclusions

This large, national-level study examined variations in UPF intake among Canadian children and adults across socio-demographic characteristics, namely age, sex, education, income adequacy, immigrant status, household food security status and region of residence. Although UPF consumption was pervasive among all socio-demographic groups, the most substantive differences in the mean energy contribution of UPF were observed by age group, immigrant status and household food security status. Because the traditional food practices of many immigrant communities in Canada are known to be healthy and low in UPF, this points to the need for efforts to slow down the process of dietary acculturation and to protect and promote these rich culinary traditions across the population at large. Our findings also suggest that policies and programmes to promote healthier eating among young persons and to curb the prevalence of household food insecurity could contribute to not only reducing UPF intake but also mitigating social inequalities in the consumption of these products. Given the overall high consumption of UPF in Canada, the results of this study support policies and interventions to promote environments favouring healthy diets and to reduce exposure to UPF among all socio-demographic segments of the population.
